# 2-*sec*-Butyl-1-(2-hy­droxy­eth­yl)-1*H*-benzimidazole-5-carboxylic acid

**DOI:** 10.1107/S1600536812023884

**Published:** 2012-06-02

**Authors:** Nurasyikin Hamzah, Nurziana Ngah, Shafida Abd Hamid, Aisyah Saad Abdul Rahim

**Affiliations:** a Kulliyyah of Science, International Islamic University Malaysia, Bandar Indera Mahkota, 25200 Kuantan, Pahang, Malaysia; b School of Pharmaceutical Sciences, Universiti Sains Malaysia, 11800 Penang, Malaysia

## Abstract

In the title compound, C_14_H_18_N_2_O_3_, the carb­oxy­lic group is tilted by 12.00 (4)° with respect to the mean plane throught the benzimidazole ring system. The alcohol and carboxyl hydroxy groups are involved in intermolecular O—H⋯O and O—H⋯N hydrogen bonds, forming a two-dimensional network extending parallel the *ab* plane. The network is further stabilized by weak C—H⋯O inter­actions. The *sec*-butyl group is disordered over two sets of sites with refined occupancies of 0.484 (4) and 0.516 (4).

## Related literature

For related structures, see: Arumugam *et al.* (2011 ▶); Hamzah *et al.* (2012 ▶). For therapeutic properties of benzimidazole derivatives, see: Xue *et al.* (2011 ▶); Gellis *et al.* (2008 ▶); Boiani *et al.* (2009 ▶). For standard bond lengths, see: Allen *et al.* (1987 ▶). For the low-temperature device used in the data collection, see: Cosier & Glazer (1986 ▶).
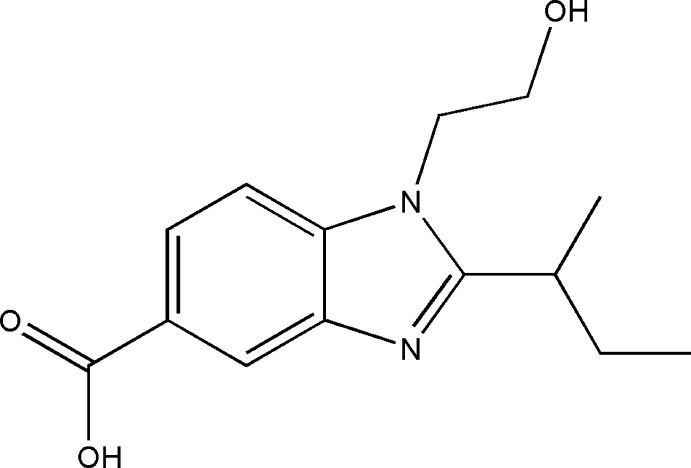


## Experimental

### 

#### Crystal data

C_14_H_18_N_2_O_3_
*M* *_r_* = 262.30
Monoclinic, 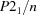

*a* = 8.9427 (2) Å
*b* = 8.2067 (2) Å
*c* = 18.3536 (3) Å
β = 94.415 (1)°
*V* = 1342.97 (5) Å^3^
*Z* = 4
Mo *K*α radiation
μ = 0.09 mm^−1^
*T* = 100 K
0.50 × 0.36 × 0.16 mm

#### Data collection

Bruker SMART APEXII CCD area-detector diffractometer
Absorption correction: multi-scan (*SADABS*; Bruker, 2009 ▶) *T*_min_ = 0.955, *T*_max_ = 0.986
11481 measured reflections
2491 independent reflections
2190 reflections with *I* > 2σ(*I*)
*R*_int_ = 0.021

#### Refinement

*R*[*F* ^2^ > 2σ(*F* ^2^)] = 0.034
*wR*(*F* ^2^) = 0.088
*S* = 1.03
2491 reflections
200 parameters
6 restraints
H atoms treated by a mixture of independent and constrained refinement
Δρ_max_ = 0.20 e Å^−3^
Δρ_min_ = −0.19 e Å^−3^

### 

Data collection: *APEX2* (Bruker, 2009 ▶); cell refinement: *SAINT* (Bruker, 2009 ▶); data reduction: *SAINT*; program(s) used to solve structure: *SHELXTL* (Sheldrick, 2008 ▶); program(s) used to refine structure: *SHELXTL*; molecular graphics: *SHELXTL*; software used to prepare material for publication: *SHELXTL* and *PLATON* (Spek, 2009 ▶).

## Supplementary Material

Crystal structure: contains datablock(s) global, I. DOI: 10.1107/S1600536812023884/rz2764sup1.cif

Structure factors: contains datablock(s) I. DOI: 10.1107/S1600536812023884/rz2764Isup2.hkl

Supplementary material file. DOI: 10.1107/S1600536812023884/rz2764Isup3.cml

Additional supplementary materials:  crystallographic information; 3D view; checkCIF report

## Figures and Tables

**Table 1:** Hydrogen-bond geometry (Å, °)

*D*—H⋯*A*	*D*—H	H⋯*A*	*D*⋯*A*	*D*—H⋯*A*
O2—H2⋯O3^i^	0.92 (2)	1.71 (2)	2.6227 (13)	175.8 (16)
O3—H3⋯N1^ii^	0.94 (2)	1.801 (19)	2.7193 (13)	165.1 (17)
C9—H9*B*⋯O1^iii^	0.99	2.43	3.2925 (15)	145
C12*X*—H12*E*⋯O1^iv^	0.98	2.55	3.517 (18)	167

Symmetry codes: (i) 
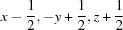
; (ii) 
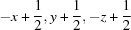
; (iii) 
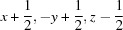
; (iv) 
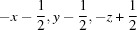
.

**Fig. 1.**
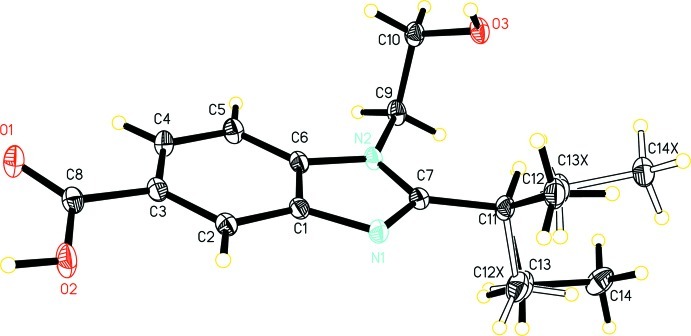
The molecular structure of the title compound, with displacement ellipsods drawn at the 50% probability level. Both disorder component are shown. Atoms labelled with suffix X denote the major component of disorder.

**Fig. 2.**
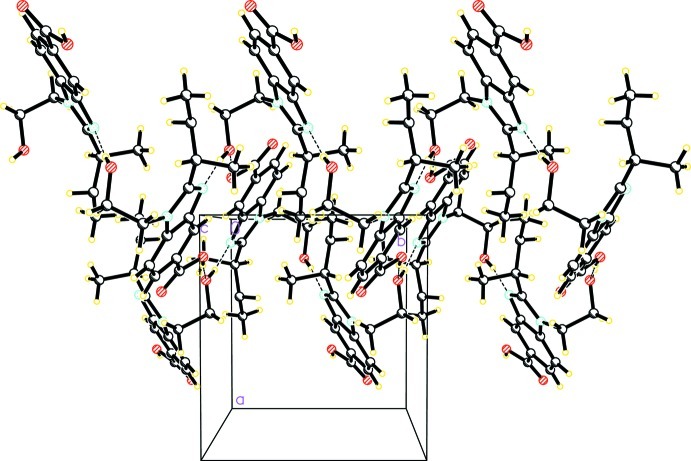
The molecular packing of the title compound viewed down the c axis. The minor disorder components have been omited for clarity.

## supplementary crystallographic information

### Comment

The synthesis and biological evaluation of benzimidazole derivatives is an active area of research in medicinal chemistry. Several papers reported some biological effects of benzimidazole derivatives against enteroviruses (Xue *et al.*, 2011), as anticancer agents (Gellis *et al.*, 2008) and anti-trypanosomatid agents (Boiani *et al.*, 2009). In continuation of our study in this field (Arumugam *et al.*, 2011; Hamzah *et al.*, 2012), the crystal structure of the title compound is described herein.

The title molecule (Fig. 1) is benzimidazole carboxylic acid derivative and is similar to other benzimidazole ethyl ester (Arumugam *et al.*, 2011; Hamzah *et al.*, 2012) derivatives. The benzimidazole ring system is essentially planar, with atom C2 deviating 0.040 (1) Å from its mean plane. The dihedral angle it forms with the carboxylic group is 12.00 (4)°. The bond lengths (Allen *et al.*, 1987) and angles are in normal ranges. The atoms of the *sec*-butyl group (C12/C13/C14) are disordered over two sets of sites, with refined occupancies of 0.484 (4) and 0.516 (4).

In the crystal structure, both hydroxy groups are involved in intermolecular O2—H2···O3 and O3—H3···N1 hydrogen bonds (Table 1) to form a two-dimensional network propagating parallel to the *ab* plane (Fig. 2). The network is further stabilized by weak C9—H9B···O1 and, C12X—H12E···O1 contacts.

### Experimental

To a solution of 2-*sec*-butyl-1-(2-hydroxy-ethyl)-1*H*-benzimidazole-5-carboxylic acid ethyl ester (136 mg, 0.47 mmol) in THF (2 ml) was added NaOH (4 N, 0.5 ml). The mixture was refluxed at 66 °C untill all reactants fully converted to the desired acid. The progress of the reaction was monitored by TLC (EtOAc/hexane 4:1 *v*/*v*). Upon completion, THF was removed under pressure and the mixture acidified using 2 M HCl to raise pH to 6–7. Removal of excess water *in vacuo* gave a white precipitate which was later dissolved in butanol to separate the carboxylic acid and the salt. The filtrate was again evaporated under reduced pressure to obtain the crude product. The title compound was recrystallized using MeOH to afford colourless single crystals.

### Refinement

X-ray data were collected at 100 K (Cosier & Glazer, 1986). Hydroxyl H-atom [O2—H2 = 0.92 (2) Å and O3—H3 = 0.94 (4) Å] were located in a difference Fourier map and refined freely. The remaining H atoms attached to C atoms were fixed geometrically and refined as riding model with C—H= 0.95–1.00 Å and with *U*_iso_(H)=1.2 or 1.5*U*_eq_(C). A rotating group model was applied to the methyl groups. The C12, C13 and C14 atoms of the *sec*-butyl group are disordered over two sites and refined with site occupancies of 0.484 (4) and 0.516 (4). The disordered atoms were refined with the C—C distances restrained to be 1.54 (1) Å.

### Crystal data

**Table:**

C_14_H_18_N_2_O_3_	*F*(000) = 560
*M**_r_* = 262.30	*D*_x_ = 1.297 Mg m^−^^3^
Monoclinic, *P*2_1_/*n*	Mo *K*α radiation, λ = 0.71073 Å
Hall symbol: -P 2yn	Cell parameters from 6388 reflections
*a* = 8.9427 (2) Å	θ = 2.4–25.5°
*b* = 8.2067 (2) Å	µ = 0.09 mm^−^^1^
*c* = 18.3536 (3) Å	*T* = 100 K
β = 94.415 (1)°	Block, colourless
*V* = 1342.97 (5) Å^3^	0.50 × 0.36 × 0.16 mm
*Z* = 4	

### Data collection

**Table:**

Bruker SMART APEXII CCD area-detector diffractometer	2491 independent reflections
Radiation source: fine-focus sealed tube	2190 reflections with *I* > 2σ(*I*)
Graphite monochromator	*R*_int_ = 0.021
Detector resolution: 83.66 pixels mm^-1^	θ_max_ = 25.5°, θ_min_ = 2.4°
φ and ω scan	*h* = −10→10
Absorption correction: multi-scan (*SADABS*; Bruker, 2009)	*k* = −9→9
*T*_min_ = 0.955, *T*_max_ = 0.986	*l* = −22→22
11481 measured reflections	

### Refinement

**Table:**

Refinement on *F*^2^	Primary atom site location: structure-invariant direct methods
Least-squares matrix: full	Secondary atom site location: difference Fourier map
*R*[*F*^2^ > 2σ(*F*^2^)] = 0.034	Hydrogen site location: inferred from neighbouring sites
*wR*(*F*^2^) = 0.088	H atoms treated by a mixture of independent and constrained refinement
*S* = 1.03	*w* = 1/[σ^2^(*F*_o_^2^) + (0.0428*P*)^2^ + 0.5356*P*] where *P* = (*F*_o_^2^ + 2*F*_c_^2^)/3
2491 reflections	(Δ/σ)_max_ < 0.001
200 parameters	Δρ_max_ = 0.20 e Å^−^^3^
6 restraints	Δρ_min_ = −0.19 e Å^−^^3^

### Special details

**Table:**

Experimental. The crystal was placed in the cold stream of an Oxford Cryosystems Cobra open=flow nitrogen cryostat (Cosier & Glazer, 1986) operating at 100.0 (1) K.
Geometry. All e.s.d.'s (except the e.s.d. in the dihedral angle between two l.s. planes) are estimated using the full covariance matrix. The cell e.s.d.'s are taken into account individually in the estimation of e.s.d.'s in distances, angles and torsion angles; correlations between e.s.d.'s in cell parameters are only used when they are defined by crystal symmetry. An approximate (isotropic) treatment of cell e.s.d.'s is used for estimating e.s.d.'s involving l.s. planes.
Refinement. Refinement of *F*^2^ against ALL reflections. The weighted *R*-factor *wR* and goodness of fit *S* are based on *F*^2^, conventional *R*-factors *R* are based on *F*, with *F* set to zero for negative *F*^2^. The threshold expression of *F*^2^ > σ(*F*^2^) is used only for calculating *R*-factors(gt) *etc*. and is not relevant to the choice of reflections for refinement. *R*-factors based on *F*^2^ are statistically about twice as large as those based on *F*, and *R*- factors based on ALL data will be even larger.

### Fractional atomic coordinates and isotropic or equivalent isotropic displacement parameters (Å^2^)

**Table:**

	*x*	*y*	*z*	*U*_iso_*/*U*_eq_	Occ. (<1)
O1	−0.34306 (10)	0.27634 (12)	0.46443 (5)	0.0256 (2)	
O2	−0.18373 (11)	0.07220 (12)	0.49284 (5)	0.0287 (2)	
O3	0.20151 (10)	0.42060 (11)	0.12007 (5)	0.0219 (2)	
N1	0.12053 (11)	0.03383 (12)	0.26410 (5)	0.0190 (2)	
N2	−0.00305 (11)	0.18127 (12)	0.17645 (5)	0.0180 (2)	
C1	−0.00646 (13)	0.10204 (14)	0.29170 (6)	0.0170 (3)	
C2	−0.05883 (13)	0.09255 (15)	0.36115 (6)	0.0180 (3)	
H2A	−0.0087	0.0282	0.3985	0.022*	
C3	−0.18717 (13)	0.18066 (15)	0.37392 (7)	0.0186 (3)	
C4	−0.26332 (14)	0.27436 (16)	0.31848 (7)	0.0211 (3)	
H4A	−0.3507	0.3330	0.3290	0.025*	
C5	−0.21368 (14)	0.28282 (16)	0.24910 (7)	0.0214 (3)	
H5A	−0.2653	0.3450	0.2114	0.026*	
C6	−0.08397 (13)	0.19556 (15)	0.23719 (6)	0.0176 (3)	
C7	0.11917 (13)	0.08457 (15)	0.19581 (6)	0.0181 (3)	
C8	−0.24608 (13)	0.18323 (15)	0.44752 (7)	0.0194 (3)	
C9	−0.03388 (14)	0.27534 (16)	0.10911 (6)	0.0205 (3)	
H9A	−0.1435	0.2911	0.1000	0.025*	
H9B	0.0017	0.2136	0.0674	0.025*	
C10	0.04292 (14)	0.44020 (16)	0.11404 (7)	0.0217 (3)	
H10A	0.0124	0.5050	0.0699	0.026*	
H10B	0.0116	0.5000	0.1572	0.026*	
C11	0.23294 (13)	0.03938 (15)	0.14321 (6)	0.0212 (3)	
H11A	0.2217	0.1164	0.1009	0.025*	0.484 (4)
H11B	0.2149	0.1112	0.0994	0.025*	0.516 (4)
C12	0.3911 (9)	0.061 (4)	0.1808 (11)	0.0279 (10)	0.484 (4)
H12A	0.4656	0.0439	0.1451	0.042*	0.484 (4)
H12B	0.4070	−0.0189	0.2204	0.042*	0.484 (4)
H12C	0.4015	0.1712	0.2010	0.042*	0.484 (4)
C13	0.208 (3)	−0.135 (2)	0.1137 (10)	0.0288 (5)	0.484 (4)
H13A	0.2441	−0.2149	0.1513	0.035*	0.484 (4)
H13B	0.0998	−0.1539	0.1012	0.035*	0.484 (4)
C14	0.2951 (4)	−0.1541 (4)	0.04528 (15)	0.0302 (8)	0.484 (4)
H14A	0.2766	−0.2628	0.0244	0.045*	0.484 (4)
H14B	0.4026	−0.1406	0.0586	0.045*	0.484 (4)
H14C	0.2616	−0.0713	0.0092	0.045*	0.484 (4)
C12X	0.202 (2)	−0.1350 (18)	0.1179 (9)	0.0288 (5)	0.516 (4)
H12D	0.2770	−0.1682	0.0847	0.043*	0.516 (4)
H12E	0.1017	−0.1412	0.0924	0.043*	0.516 (4)
H12F	0.2071	−0.2077	0.1604	0.043*	0.516 (4)
C13X	0.3932 (8)	0.068 (4)	0.1744 (10)	0.0279 (10)	0.516 (4)
H13C	0.4247	−0.0221	0.2081	0.033*	0.516 (4)
H13D	0.3986	0.1709	0.2025	0.033*	0.516 (4)
C14X	0.4982 (3)	0.0770 (3)	0.11315 (15)	0.0282 (8)	0.516 (4)
H14D	0.6015	0.0908	0.1341	0.042*	0.516 (4)
H14E	0.4702	0.1698	0.0814	0.042*	0.516 (4)
H14F	0.4905	−0.0238	0.0845	0.042*	0.516 (4)
H3	0.248 (2)	0.459 (2)	0.1644 (11)	0.055 (5)*	
H2	−0.224 (2)	0.080 (2)	0.5373 (11)	0.057 (6)*	

### Atomic displacement parameters (Å^2^)

**Table:**

	*U*^11^	*U*^22^	*U*^33^	*U*^12^	*U*^13^	*U*^23^
O1	0.0245 (5)	0.0349 (5)	0.0183 (5)	0.0060 (4)	0.0066 (4)	−0.0005 (4)
O2	0.0299 (5)	0.0407 (6)	0.0167 (5)	0.0106 (4)	0.0103 (4)	0.0073 (4)
O3	0.0202 (5)	0.0288 (5)	0.0172 (5)	−0.0024 (4)	0.0056 (4)	−0.0024 (4)
N1	0.0204 (5)	0.0202 (5)	0.0170 (5)	0.0017 (4)	0.0062 (4)	0.0013 (4)
N2	0.0179 (5)	0.0223 (5)	0.0143 (5)	0.0006 (4)	0.0046 (4)	0.0019 (4)
C1	0.0165 (6)	0.0178 (6)	0.0169 (6)	−0.0027 (5)	0.0034 (4)	−0.0007 (5)
C2	0.0187 (6)	0.0193 (6)	0.0162 (6)	−0.0019 (5)	0.0024 (5)	0.0008 (5)
C3	0.0179 (6)	0.0218 (6)	0.0166 (6)	−0.0036 (5)	0.0035 (5)	−0.0015 (5)
C4	0.0172 (6)	0.0264 (7)	0.0203 (6)	0.0016 (5)	0.0050 (5)	−0.0005 (5)
C5	0.0184 (6)	0.0273 (7)	0.0185 (6)	0.0016 (5)	0.0023 (5)	0.0030 (5)
C6	0.0177 (6)	0.0204 (6)	0.0150 (6)	−0.0034 (5)	0.0038 (5)	−0.0005 (5)
C7	0.0191 (6)	0.0186 (6)	0.0171 (6)	−0.0006 (5)	0.0043 (5)	0.0008 (5)
C8	0.0164 (6)	0.0249 (6)	0.0170 (6)	−0.0026 (5)	0.0019 (5)	−0.0012 (5)
C9	0.0184 (6)	0.0294 (7)	0.0139 (6)	0.0021 (5)	0.0026 (5)	0.0038 (5)
C10	0.0227 (6)	0.0254 (7)	0.0177 (6)	0.0047 (5)	0.0059 (5)	0.0042 (5)
C11	0.0235 (6)	0.0243 (6)	0.0168 (6)	0.0051 (5)	0.0077 (5)	0.0030 (5)
C12	0.0227 (7)	0.033 (2)	0.029 (2)	−0.0012 (6)	0.0104 (7)	−0.006 (2)
C13	0.0250 (15)	0.0337 (8)	0.0282 (15)	0.0024 (7)	0.0053 (10)	−0.0113 (9)
C14	0.0394 (17)	0.0299 (16)	0.0210 (15)	0.0092 (13)	0.0008 (12)	−0.0061 (12)
C12X	0.0250 (15)	0.0337 (8)	0.0282 (15)	0.0024 (7)	0.0053 (10)	−0.0113 (9)
C13X	0.0227 (7)	0.033 (2)	0.029 (2)	−0.0012 (6)	0.0104 (7)	−0.006 (2)
C14X	0.0217 (14)	0.0327 (15)	0.0313 (15)	0.0008 (11)	0.0089 (11)	0.0010 (12)

### Geometric parameters (Å, º)

**Table:**

O1—C8	1.2140 (15)	C10—H10B	0.9900
O2—C8	1.3273 (16)	C11—C13X	1.520 (11)
O2—H2	0.92 (2)	C11—C12X	1.523 (11)
O3—C10	1.4231 (15)	C11—C12	1.535 (12)
O3—H3	0.94 (2)	C11—C13	1.537 (12)
N1—C7	1.3199 (16)	C11—H11A	1.0000
N1—C1	1.3958 (15)	C11—H11B	1.0000
N2—C7	1.3750 (16)	C12—H12A	0.9800
N2—C6	1.3799 (15)	C12—H12B	0.9800
N2—C9	1.4653 (15)	C12—H12C	0.9800
C1—C2	1.3936 (17)	C13—C14	1.536 (12)
C1—C6	1.4013 (17)	C13—H13A	0.9900
C2—C3	1.3916 (17)	C13—H13B	0.9900
C2—H2A	0.9500	C14—H14A	0.9800
C3—C4	1.4084 (18)	C14—H14B	0.9800
C3—C8	1.4873 (16)	C14—H14C	0.9800
C4—C5	1.3822 (17)	C12X—H12D	0.9800
C4—H4A	0.9500	C12X—H12E	0.9800
C5—C6	1.3947 (17)	C12X—H12F	0.9800
C5—H5A	0.9500	C13X—C14X	1.521 (11)
C7—C11	1.5019 (16)	C13X—H13C	0.9900
C9—C10	1.5169 (18)	C13X—H13D	0.9900
C9—H9A	0.9900	C14X—H14D	0.9800
C9—H9B	0.9900	C14X—H14E	0.9800
C10—H10A	0.9900	C14X—H14F	0.9800
			
C8—O2—H2	109.3 (12)	C13X—C11—C12X	113.8 (16)
C10—O3—H3	113.7 (12)	C7—C11—C12	109.2 (7)
C7—N1—C1	105.66 (10)	C12X—C11—C12	112.6 (16)
C7—N2—C6	107.23 (9)	C7—C11—C13	111.6 (6)
C7—N2—C9	128.04 (10)	C13X—C11—C13	112.5 (15)
C6—N2—C9	124.07 (10)	C12—C11—C13	111.6 (16)
C2—C1—N1	130.52 (11)	C7—C11—H11A	108.1
C2—C1—C6	120.07 (11)	C13X—C11—H11A	103.3
N1—C1—C6	109.39 (10)	C12X—C11—H11A	110.6
C3—C2—C1	117.68 (11)	C12—C11—H11A	108.1
C3—C2—H2A	121.2	C13—C11—H11A	108.1
C1—C2—H2A	121.2	C7—C11—H11B	107.2
C2—C3—C4	121.41 (11)	C13X—C11—H11B	107.5
C2—C3—C8	120.98 (11)	C12X—C11—H11B	107.1
C4—C3—C8	117.58 (11)	C12—C11—H11B	112.3
C5—C4—C3	121.42 (11)	C13—C11—H11B	104.7
C5—C4—H4A	119.3	C11—C12—H12A	109.5
C3—C4—H4A	119.3	C11—C12—H12B	109.5
C4—C5—C6	116.65 (11)	C11—C12—H12C	109.5
C4—C5—H5A	121.7	C14—C13—C11	108.4 (12)
C6—C5—H5A	121.7	C14—C13—H13A	110.0
N2—C6—C5	131.64 (11)	C11—C13—H13A	110.0
N2—C6—C1	105.60 (10)	C14—C13—H13B	110.0
C5—C6—C1	122.75 (11)	C11—C13—H13B	110.0
N1—C7—N2	112.11 (10)	H13A—C13—H13B	108.4
N1—C7—C11	125.12 (11)	C11—C12X—H12D	109.5
N2—C7—C11	122.74 (10)	C11—C12X—H12E	109.5
O1—C8—O2	123.08 (11)	H12D—C12X—H12E	109.5
O1—C8—C3	123.44 (11)	C11—C12X—H12F	109.5
O2—C8—C3	113.48 (10)	H12D—C12X—H12F	109.5
N2—C9—C10	111.39 (10)	H12E—C12X—H12F	109.5
N2—C9—H9A	109.3	C11—C13X—C14X	110.3 (11)
C10—C9—H9A	109.3	C11—C13X—H13C	109.6
N2—C9—H9B	109.3	C14X—C13X—H13C	109.6
C10—C9—H9B	109.3	C11—C13X—H13D	109.6
H9A—C9—H9B	108.0	C14X—C13X—H13D	109.6
O3—C10—C9	110.35 (10)	H13C—C13X—H13D	108.1
O3—C10—H10A	109.6	C13X—C14X—H14D	109.5
C9—C10—H10A	109.6	C13X—C14X—H14E	109.5
O3—C10—H10B	109.6	H14D—C14X—H14E	109.5
C9—C10—H10B	109.6	C13X—C14X—H14F	109.5
H10A—C10—H10B	108.1	H14D—C14X—H14F	109.5
C7—C11—C13X	112.7 (7)	H14E—C14X—H14F	109.5
C7—C11—C12X	108.1 (6)		
			
C7—N1—C1—C2	−178.32 (12)	C9—N2—C7—C11	−10.00 (19)
C7—N1—C1—C6	0.04 (13)	C2—C3—C8—O1	168.54 (12)
N1—C1—C2—C3	177.08 (12)	C4—C3—C8—O1	−9.59 (18)
C6—C1—C2—C3	−1.13 (17)	C2—C3—C8—O2	−12.30 (16)
C1—C2—C3—C4	0.97 (18)	C4—C3—C8—O2	169.57 (11)
C1—C2—C3—C8	−177.09 (11)	C7—N2—C9—C10	−84.32 (15)
C2—C3—C4—C5	−0.06 (19)	C6—N2—C9—C10	85.16 (14)
C8—C3—C4—C5	178.06 (11)	N2—C9—C10—O3	64.65 (12)
C3—C4—C5—C6	−0.67 (18)	N1—C7—C11—C13X	−51.4 (13)
C7—N2—C6—C5	177.59 (13)	N2—C7—C11—C13X	130.8 (13)
C9—N2—C6—C5	6.3 (2)	N1—C7—C11—C12X	75.2 (10)
C7—N2—C6—C1	−1.03 (13)	N2—C7—C11—C12X	−102.5 (10)
C9—N2—C6—C1	−172.36 (11)	N1—C7—C11—C12	−47.6 (13)
C4—C5—C6—N2	−177.92 (12)	N2—C7—C11—C12	134.7 (13)
C4—C5—C6—C1	0.50 (18)	N1—C7—C11—C13	76.3 (11)
C2—C1—C6—N2	179.18 (11)	N2—C7—C11—C13	−101.4 (11)
N1—C1—C6—N2	0.62 (13)	C7—C11—C13—C14	163.5 (10)
C2—C1—C6—C5	0.41 (18)	C13X—C11—C13—C14	−68.6 (16)
N1—C1—C6—C5	−178.15 (11)	C12X—C11—C13—C14	179 (100)
C1—N1—C7—N2	−0.72 (14)	C12—C11—C13—C14	−73.9 (18)
C1—N1—C7—C11	−178.65 (11)	C7—C11—C13X—C14X	−160.6 (13)
C6—N2—C7—N1	1.13 (14)	C12X—C11—C13X—C14X	76 (2)
C9—N2—C7—N1	172.02 (11)	C12—C11—C13X—C14X	153 (29)
C6—N2—C7—C11	179.12 (11)	C13—C11—C13X—C14X	72 (2)

### Hydrogen-bond geometry (Å, º)

**Table:**

*D*—H···*A*	*D*—H	H···*A*	*D*···*A*	*D*—H···*A*
O2—H2···O3^i^	0.92 (2)	1.71 (2)	2.6227 (13)	175.8 (16)
O3—H3···N1^ii^	0.94 (2)	1.801 (19)	2.7193 (13)	165.1 (17)
C9—H9*B*···O1^iii^	0.99	2.43	3.2925 (15)	145
C12*X*—H12*E*···O1^iv^	0.98	2.55	3.517 (18)	167

Symmetry codes: (i) *x*−1/2, −*y*+1/2, *z*+1/2; (ii) −*x*+1/2, *y*+1/2, −*z*+1/2; (iii) *x*+1/2, −*y*+1/2, *z*−1/2; (iv) −*x*−1/2, *y*−1/2, −*z*+1/2.
